# Delayed Diagnosis of Acute Q Fever, China

**DOI:** 10.3201/eid2812.221118

**Published:** 2022-12

**Authors:** Dan Li, Hui Liu, Ming Liu, Caiyun Chang, Xiaodong Zhao, Hao Yu, Lina Yan, Huiju Han, Xue-jie Yu

**Affiliations:** State Key Laboratory of Virology, School of Public Health, Wuhan University, Wuhan, China (D. Li, L. Yan, H. Han, X.-j. Yu);; Jinan Center for Disease Control and Prevention, Jinan, China (H. Liu, M. Liu, C. Chang, X. Zhao);; University of Texas MD Anderson Cancer Center, Houston, Texas, USA (H. Yu)

**Keywords:** acute Q fever, China, zoonoses, Coxiella burnetii, bacteria

## Abstract

We report a patient in China with fever of unknown origin who visited 3 hospitals in 3 weeks and was finally given a diagnosis of acute Q fever, determined by metagenomics next-generation sequencing. Our results indicate that physicians are unfamiliar with Q fever and the disease is neglected in China.

Q fever is an important worldwide zoonosis with nonspecific symptoms, making diagnosis challenging (*1*–*3*). Humans become infected mainly by inhalation of *Coxiella burnetii*–contaminated aerosols from animal waste or contaminated soil (*4*). *C. burnetii* is listed as a biologic weapon in the United States, and Q fever is a nationally notifiable disease in the United States, Australia, Netherlands, and Japan, but it is not a notifiable disease in China (*2*,*5*–*7*). Serologic epidemiology indicates that *C. burnetii* is widely distributed in China, but Q fever is rarely reported and might be neglected (*2*,*7*). We report a case of Q fever in a man in Shandong Province, China. The need for ethics approval and informed consent was waived, granted by the National Health Commission of China as part of outbreak investigation of infectious disease.

A 55-year-old man in a rural area of Jinan, Shandong Province, China, had fever (38.3 °C), headache, fatigue, loss of appetite, and myalgia develop on August 24, 2019 (Figure 1). He visited a local town hospital and was treated with acetaminophen and chlorpheniramine. When his symptoms persisted, he visited a county hospital on the 9th day after illness onset with a body temperature of 38.5°C and was treated with oral cefprozil and levofloxacin. On the 14th day of illness, with no improvement of his symptoms, he was transferred to a local municipal hospital. At admission, his body temperature was 39.0°C. Blood tests revealed elevation in neutrophil count and ratio, C-reactive protein, serum procalcitonin, and liver enzymes (Table). Bacterial culture showed no growth of microorganisms in either aerobic or anaerobic cultures (BD BACTEC FX 200 blood culture instrument, https://www.bd.com). We used PCR or immunologic test kits to test for viruses (influenza virus, severe fever with thrombocytopenia syndrome virus, Hantan virus, hepatitis B, hepatitis C, Epstein-Barr, and cytomegalovirus) and bacteria (*Brucella*, *Mycobacterium tuberculosis*, typhoid, and paratyphoid). We observed no positive results.

**Figure 1:**
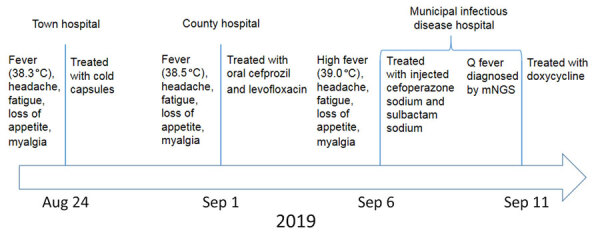
Timeline of illness in a patient with Q fever in Shandong Province, China, 2019. mNGS, metagenomics next-generation sequencing.

**Table:** Blood and biochemical indicators for a patient with Q fever, Shandong Province, China, 2019

Category	Value	Reference range
Neutrophil count, × 10^9^ cells/L	8.30	2.00–7.00
Neutrophils, %	86.20	50.00–70.00
Leukocyte, × 10^9^ cells/L	9.64	4–10
Leukomonocyte, × 10^9^ cells/L	0.79	0.80–4.0
Platelet, × 10^9^/L	210	100–300
Erythrocytes, × 10^12^ cells/L	4.13	4.0–5.5
C-reactive protein, mg/L	21.96	0.068–8.20
Serum procalcitonin, ng/mL	2.50	0–0.05
Alanine aminotransferase, U/L	99	0–40
Alkaline phosphatase, U/L	208	40–150
Aspartate transaminase, U/L	51	0–40
Gamma-glutamyl transpeptidase, U/L	333	12–64

We sent samples to the CapitalBio MedLab in Beijing, China, where metagenomics next-generation sequencing (mNGS) was performed to determine the etiologic agent (Ion Proton Sequencer, https://www.thermofisher.com). A blood sample obtained from the patient provided DNA for that analysis (QIAamp MinElute ccfDNA Mini Kit; https://www.qiagen.com). On the 19th day after illness onset, the mNGS result revealed *C. burnetii* sequences in the patient’s blood sample; no other pathogens were observed. The sequence coverage rate of the *C. burnetii* genome was 97.66% (2,078,829 bp) with 137,272 reads (average length 141 bp, average quality 23), 1,105 contigs (range 262–16,242 bp), and an estimated 1.80 × 10^4^ copies/mL of *C. burnetii* in the sequencing sample. The mNGS result clearly indicated that the patient was infected with *C. burnetii*. Phylogenetic analysis revealed that the isocitrate dehydrogenase sequence from the patient formed a monophyletic group with sequences of *C. burnetii* from goats and from humans diagnosed with acute Q fever from GenBank (Figure 2). The isocitrate dehydrogenase sequence homology between the patient and those sequences were 99.85%–99.92%. 

**Figure 2:**
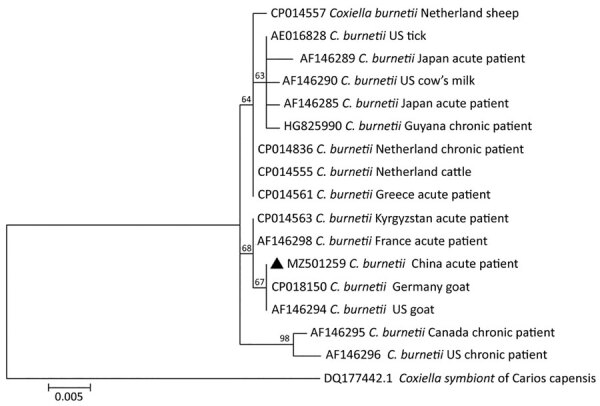
Phylogenetic tree of *Coxiella burnetii* from a patient with Q fever in Shandong Province, China, 2019. Triangle indicates the strain detected in this study. The phylogenetic tree was constructed using the complete isocitrate dehydrogenase gene sequence (1,300-bp) with the maximum-likelihood method using MEGA 7.0 (https://www.megasoftware.net). Bootstrap values >50% from 1,000 replicates (shown on the nodes). Scale bar indicates substitutions per site.

We performed cardiac ultrasound of the patient, which showed normal cardiopulmonary function and ruled out Q fever endocarditis. We treated the patient with oral doxycycline (100 mg 2×/d). His symptoms disappeared in 1 week, and he was discharged and continued on oral doxycycline (100 mg 2×/d) for 1 more week. We followed the patient for 1 year, noting no recurrence of Q fever.

This patient worked as a woodworker in a village without nearby abattoirs. He did not raise animals, but there were goats in his village, and mice were often observed around his living and working places. He denied any contact with domesticated or wild animals, ingestion of unpasteurized dairy products or uncooked meat, tick bite, exposure to similar patients, or any travel history to other places in China or abroad in the months before his illness.

Multiple factors likely delayed diagnosis of this patient with Q fever. Although nonspecific symptoms contributed, the greatest obstacles to diagnosis, we believe, were unawareness of the existence of Q fever by physicians and lack of conventional diagnostic reagents of Q fever, such as serologic and *C. burnetii*–specific PCR reagents, in the medical institutions our patient visited (*8*,*9*).

In conclusion, we report a patient with febrile illness from Shandong Province, China, without etiologic diagnosis and appropriate treatment for 3 weeks, until mNGS revealed *C. burnetii* genomic sequences in the patient’s blood. Our study suggests that physicians need to be more aware that Q fever is widespread in China and should be considered when diagnosing patients with persistent fever of unknown origin, even without clear exposure history. In addition, conventional diagnostic reagents of Q fever should be stored in local medical institutions in China. mNGS is a method to randomly sequence all nucleic acids and identify organisms by bioinformatics analysis in a sample, which is useful in identifying unknown pathogens. Our case supports previous studies that demonstrated that mNGS can be used to diagnose Q fever and other pathogens in humans (*10*).

## References

[1] Derrick EH. “Q” fever, a new fever entity: clinical features, diagnosis and laboratory investigation. Rev Infect Dis. 1983;5:790–800. 10.1093/clinids/5.4.790 6622891

[2] El-Mahallawy HS, Lu G, Kelly P, Xu D, Li Y, Fan W, et al. Q fever in China: a systematic review, 1989-2013. Epidemiol Infect. 2015;143:673–81. 10.1017/S0950268814002593 25274488 PMC9507106

[3] Karageorgou I, Kogerakis N, Labropoulou S, Hatzianastasiou S, Mentis A, Stavridis G, et al. Q fever endocarditis and a new genotype of *Coxiella burnetii*, Greece. Emerg Infect Dis. 2020;26:2527–9. 10.3201/eid2610.191616 32946732 PMC7510691

[4] Parker NR, Barralet JH, Bell AM. Q fever. Lancet. 2006;367:679–88. 10.1016/S0140-6736(06)68266-4 16503466

[5] Madariaga MG, Rezai K, Trenholme GM, Weinstein RA. Q fever: a biological weapon in your backyard. Lancet Infect Dis. 2003;3:709–21. 10.1016/S1473-3099(03)00804-1 14592601

[6] Devaux CA, Osman IO, Million M, Raoult D. *Coxiella burnetii* in dromedary camels (*Camelus dromedarius*): a possible threat for humans and livestock in North Africa and the Near and Middle East? Front Vet Sci. 2020;7:558481. 10.3389/fvets.2020.558481 33251255 PMC7674558

[7] Huang M, Ma J, Jiao J, Li C, Chen L, Zhu Z, et al. The epidemic of Q fever in 2018 to 2019 in Zhuhai city of China determined by metagenomic next-generation sequencing. PLoS Negl Trop Dis. 2021;15:e0009520. 10.1371/journal.pntd.0009520 34264939 PMC8282036

[8] Stein A, Raoult D. Detection of *Coxiella burnetti* by DNA amplification using polymerase chain reaction. J Clin Microbiol. 1992;30:2462–6. 10.1128/jcm.30.9.2462-2466.1992 1401016 PMC265524

[9] Anderson A, Bijlmer H, Fournier PE, Graves S, Hartzell J, Kersh GJ, et al. Diagnosis and management of Q fever—United States, 2013: recommendations from CDC and the Q Fever Working Group. MMWR Recomm Rep. 2013;62(RR-03):1–30. 23535757

[10] Simner PJ, Miller S, Carroll KC. Understanding the promises and hurdles of metagenomic next-generation sequencing as a diagnostic tool for infectious diseases. Clin Infect Dis. 2018;66:778–88. 10.1093/cid/cix881 29040428 PMC7108102

